# Establishment and Validation of a Multivariate Predictive Scoring Model for Intravenous Immunoglobulin-Resistant Kawasaki Disease: A Study of Children From Two Centers in China

**DOI:** 10.3389/fcvm.2022.883067

**Published:** 2022-04-27

**Authors:** Changjian Li, Shu Wu, Yuanyuan Shi, Ying Liao, Yan Sun, Hui Yan, Qingyou Zhang, Jia Fu, Dan Zhou, Yong Zhang, Hongfang Jin, Junbao Du

**Affiliations:** ^1^Department of Pediatrics, Peking University First Hospital, Beijing, China; ^2^Department of Cardiology, Wuhan Children’s Hospital (Wuhan Maternal and Child Healthcare Hospital), Tongji Medical College, Huazhong University of Science and Technology, Wuhan, China; ^3^Department of General Medicine, Wuhan Fourth Hospital, Puai Hospital, Tongji Medical College, Huazhong University of Science and Technology, Wuhan, China; ^4^Key Laboratory of Molecular Cardiovascular Sciences, The Ministry of Education, Beijing, China

**Keywords:** Kawasaki disease, intravenous immunoglobulin-resistant, predictive scoring model, binary logistic regression, prognostic nutritional index

## Abstract

**Background:**

Early identification of intravenous immunoglobulin (IVIG)-resistant Kawasaki disease (KD) is important for making a suitable therapeutic strategy for children with KD.

**Methods:**

This study included a training set and an external validation set. The training set included 635 children (588 IVIG-sensitive and 47 IVIG-resistant KD) hospitalized in Wuhan Children’s Hospital, Hubei, China. Univariate analyses and binary logistic regression equation was incorporated to find the associated variables of the IVIG-resistant KD. A scoring model for predicting IVIG-resistant KD was established according to odds ratio (OR) values and receiver operating characteristic curves. The external validation set consisted of 391 children (358 IVIG-sensitive and 33 IVIG-resistant KD) hospitalized in Peking University First Hospital, Beijing, China. The predictive ability of the model of IVIG-resistant KD were externally validated by the real clinically diagnosed KD cases.

**Results:**

Fifteen variables in the training set were statistically different between IVIG-sensitive and IVIG-resistant KD children, including rash, duration of fever, peripheral blood neutrophil-to-lymphocyte ratio (NLR), prognostic nutritional index (PNI), percentage of monocytes and percentage of eosinophils, and serum alanine aminotransferase, aspartate aminotransferase, total bilirubin (TB), direct bilirubin, glutamyl transpeptidase, prealbumin, sodium ion, potassium ion and high-sensitivity C-reactive protein. According to logistic equation analysis, the final three independent correlates to IVIG-resistant KD were serum TB ≥ 12.8 μmol/L, peripheral blood NLR ≥ 5.0 and peripheral blood PNI ≤ 52.4. According to the OR values, three variables were assigned the points of 2, 2 and 1, respectively. When the score was ≥ 3 points, the sensitivity to predict IVIG-resistant KD was 80.9% and the specificity was 77.6%. In the validation set, the sensitivity, specificity and accuracy of the predictive model of IVIG-resistant KD were 72.7%, 84.9%, and 83.9%, respectively.

**Conclusion:**

A scoring model was constructed to predict IVIG-resistant KD, which would greatly assist pediatricians in the early prediction of IVIG-resistant KD.

## Introduction

Kawasaki disease (KD) is an acute vasculitic disease, the pathogenesis of which has not yet been clear (1). In particular, coronary artery lesion (CAL) is a complication that severely affects the prognosis of KD and is a primary reason for acquired heart defects in some children (2). In severe cases, KD may even be complicated with cardiac tamponade and giant coronary artery aneurysms (3, 4). With the use of intravenous immunoglobulin (IVIG), however, the incidence of CAL has decreased significantly (3, 5). Nevertheless, 10 to 20% of children with KD did not have a satisfactory control of the fever after receiving the initial standard dose of IVIG, and this group of children was called IVIG non-responsive or IVIG-resistant KD (1, 6, 7). Importantly, the pathological process of vasculitis could not be interrupted and relieved in time due to the persistence of a high inflammatory response in IVIG-resistant children, which leads to a significantly higher incidence of CAL in these children on the one hand (8), and increases the cost of hospitalization on the other.

Several studies have shown that the initial IVIG combined with corticosteroids could decrease the occurrence of IVIG resistance and CAL (9, 10). However, some studies reported that corticosteroids could increase the occurrence of CAL (11), and the treatment of corticosteroids as a second-line treatment for KD was highly controversial (12–14). The Italian Society of Pediatrics recently stated that corticosteroids could be used in combination with the initial administration of IVIG in “high-risk” children (15). This provided the possibility of concomitant corticosteroids at the time of initial IVIG in IVIG-resistant children, and therefore, further studies are needed to predict this subset of children with KD who may be resistant to IVIG and thus to evaluate in advance whether to give corticosteroids, which may be essential to improve the prognosis of the children. In fact, there were many predictive measures associated with IVIG non-response, the Japanese Kobayashi score, Egami score and Sato score systems, for instance (16–18).

Currently, a Japanese team used the IVIG-resistance prediction model in clinical and some prospective multicenter clinical control studies (9, 19). However, there was a significant decrease in the sensitivity of the Japanese scoring model across countries and regions (20, 21). There were also some studies on IVIG-resistance prediction models in China (22–26), but some models suffered from low sensitivity and specificity, lack of validation, or relatively small sample size. Thus, it is extremely necessary to establish a scoring model with a strong predictive power based on a training and an external validation design in China.

Therefore, this study was undertaken to explore a useful predictive scoring model of IVIG-resistant KD to help pediatricians in implementing proper assessment and treatment strategy for children with KD.

## Materials and Methods

### Subjects

**FIGURE 1:**
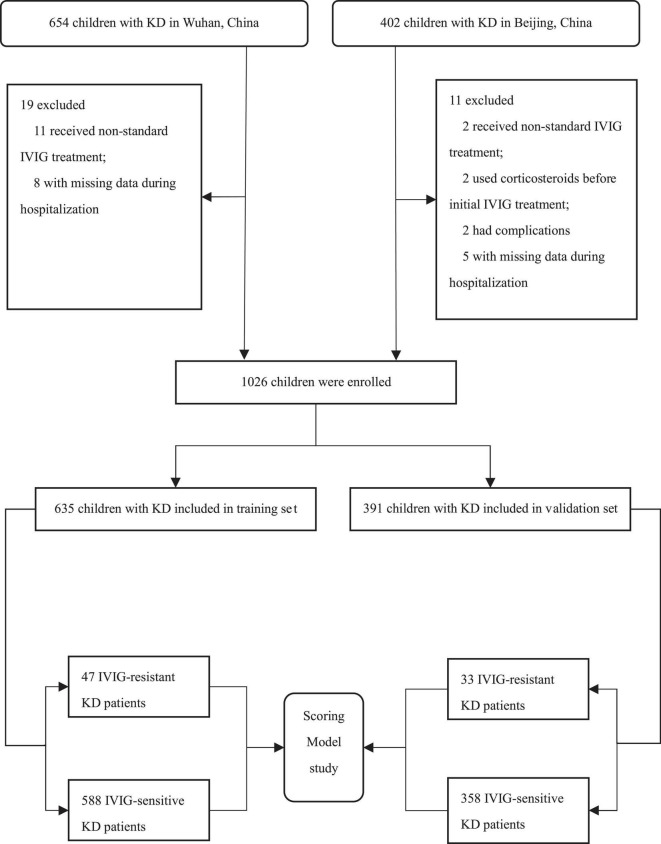
Flow chart of study subject inclusion. IVIG, intravenous immunoglobulin; KD, Kawasaki disease.

We screened 1056 patients with KD, of whom 1026 (97.2%) were eligible (Figure 1). The study consisted a training set and an external validation set. In the training set, 635 children hospitalized in Wuhan Children’s Hospital were included from January 2018 to October 2019. Of whom, 588 children (388 males and 200 females) suffered from IVIG-sensitive KD and another 47 children (30 males and 17 females) suffered from IVIG-resistant KD. Their median ages were 23.0 (12.0, 42.0) months for children with IVIG-sensitive KD and 22.0 (12.0, 39.0) months for those with IVIG-resistant KD, respectively. In addition, 391 patients hospitalized in the Department of Pediatrics, Peking University First Hospital between January 2008 and October 2019 were enrolled in the validation set. Among them, 358 children (226 males and 132 females) were IVIG-sensitive KD and 33 children (26 males and 7 females) were IVIG-resistant KD. Their median ages were 22.0 (12.0, 39.0) months for children with IVIG-sensitive KD, and 34.0 (14.5, 53.0) months for those with IVIG-resistant KD, respectively.

Criteria for the diagnosis of classic KD (CKD): CKD was classified as containing 5 or more of the 6 clinical characteristics; or the existence of 4 clinical characteristics, excluding fever caused by other diseases or rash diseases, presence of coronary artery dilatation. Six of these clinical characteristics were as below: (1) fever; (2) conjunctival congestion; (3) orofacial lesions: red lips, strawberry tongue; (4) rash; (5) peripheral limb changes: redness and swelling of palms and feet in the initial phase, and peeling of skin at the ends of limbs in the recovery phase; and (6) non-suppurative cervical lymphadenitis (27, 28).

Criteria for the diagnosis of incomplete KD (IKD): IKD was characterized by the existence of 3 of the 6 clinical characteristics and combined with CAL; or only 3 or 4 major clinical characteristics without CAL, but with one of the “other important clinical features”. Other important clinical features were referred to the diagnostic guidelines in the United States and Japan (1, 27, 28).

Criteria for diagnosis of IVIG-resistant KD: IVIG-resistant KD was defined according to the KD diagnostic criteria and having fever or re-fever after 36 h of initial IVIG treatment (1, 27, 29).

Inclusion criteria: children hospitalized with CKD or IKD had their blood routine examination and biochemistry indicator tests completed within 2 days prior to treatment with IVIG; IVIG and aspirin were routinely administered in accordance with guideline criteria (1).

Exclusion criteria: children received non-standard IVIG treatment; use of corticosteroids or other immunosuppressive drugs before the initial IVIG treatment; use of corticosteroids or other immunosuppressive drugs at the same time as the initial IVIG treatment; presence of other serious complications such as macrophage activation syndrome, shock, severe infection, septic lesions, multi-organ dysfunction, hemophagocytic syndrome, etc.; and some clinical data missing during hospitalization.

### Data Collection

The medical records of the study subjects were obtained from the electronic case system (Donghua, Beijing, China and Kaihua, Beijing, China). The information included the demographic data, clinical characteristics and the laboratory results. The demographic data included hospitalization date, hospitalization number, sex, age and body mass index (BMI). Clinical features included CKD symptoms (duration of fever before initial IVIG, conjunctival congestion, red lips/strawberry tongue, enlarged lymph nodes, rash, stiff hands and feet), frequency and dose of IVIG, presence of complications (macrophage activation syndrome, shock, severe infections, combined septic lesions, multi-organ dysfunction, hemophagocytic syndrome) and other underlying diseases (malnutrition, flat blood, liver disease, other autoimmune diseases, etc.). Laboratory test results included routine blood test results, biochemical indicators (liver function, cardiac enzymes and electrolytes), infection indicators (high-sensitivity C-reactive protein, hsCRP), and cardiac ultrasound (marking coronary artery diameter and *Z*-value) at the acute stage of KD (usually no more than 10 days). The above medical records were recorded by a dedicated staff and carefully proofread by another professional.

The study was approved by the Ethics Committee of Wuhan Children’s Hospital (2022R007–E01) and Peking University First Hospital (2020–108), and a waiver of the informed consent was granted.

### Treatment Protocol

Immediately after the initial diagnosis of CKD or IKD was established, all patients were given standard treatment for KD, including IVIG (2 g/kg) and oral aspirin (30–50 mg/kg/day) in 3 divided doses (1, 30). A downward adjustment of the aspirin to a low dose of 3–5 mg/kg/day after 48 to 72 h of fever resolution in children was done and maintained for 6–8 weeks in the absence of CAL. For children who developed CAL, aspirin was discontinued until the CAL recovered (1).

### Laboratory Tests

Blood routine examination, biochemical indicators and hsCRP were performed as follows. At the child’s first admission to the hospital prior to the initial use of IVIG, 2.0 mL of venous blood was obtained with an EDTA–K2 anticoagulated vacuum collection tube and mixed upside down and sent to the laboratory for testing. A fully automated hematology analyzer XN–3000 (Sysmex, Kobe, Japan) and BC–6800 plus (Mindray, Shenzhen, China) was used to measure blood routine examination. At the same time, 2.0 mL of venous blood was collected and centrifuged using a BY–600A centrifuge (Baiyang, Beijing, China) at 3300 *g* for 5 min. The non-hemolyzed serum was obtained, generally using a Cobas–8000 automatic biochemical analyzer (Roche, Mannheim, Germany) for biochemical parameters and a BNII auto-analyzer system (Siemens, Erlangen, Germany) for hsCRP. All specimens were tested on the machine within 2 h in the laboratory department, and the results were verified by a dedicated staff in the laboratory. Then, the blood routine, liver function, electrolytes, cardiac enzymes and hsCRP were uploaded to the electronic medical record system. Our fully automated testers were regularly calibrated and accuracy tested with coefficients of variation within normal limits. In particular, prognostic nutritional index (PNI) = albumin (ALB, g/L) + 5 × absolute value of lymphocytes (×10^9^/L) (31) and neutrophil-to-lymphocyte ratio (NLR) = neutrophils (×10^9^/L)/lymphocytes (×10^9^/L).

### Statistical Method

We used SPSS 23.0 (IBM, New York, NY, United States) for the data analysis. If the data of continuous variables in both the IVIG-sensitive and IVIG-resistant groups obeyed a normal distribution (Shapiro–Wilk test), the difference was compared by *t*-test, otherwise by the Mann–Whitney *U* test. The chi-square test was performed to compare the difference in dichotomous variables. Variables with statistical differences between groups were added to the multifactorial analysis (*P* < 0.05). Covariance diagnosis was performed and covariates were excluded before entering the multifactorial analysis. And continuous variables were transformed into dichotomous variables for an easy use. Finally, the above statistically different dichotomous variables were included into a binary logistic regression equation, and independent associated factors were obtained by stepwise regression using the backward conditional method. *P* > 0.05 for the Hosmer-Lemeshow test suggested a good fit of the equation. And finally, a predictive scoring model was established by assigning the scores by the odds ratio (OR) values of each independent risk factor, and the total points of the assignment were summarized for each child. The receiver operating characteristic (ROC) curve was performed to calculate the maximum Youden index corresponding to the cutoff value, sensitivity and specificity of the total score. Finally, in external validation set, scores were computed separately for each child, and a four-grid table was constructed based on the true clinical diagnosis and the cutoff value of the predictive scoring model to validate the sensitivity, specificity and accuracy of the model. Statistical differences were set at *P* < 0.05.

## Results

### Demographic Features and Clinical Manifestations Between Intravenous Immunoglobulin-Sensitive and Intravenous Immunoglobulin-Resistant Kawasaki Disease Children in Training Set

In the training set, no statistical differences were found in gender, age, height, weight, and BMI between IVIG-sensitive and IVIG-resistant KD children (*P* > 0.05). The clinical manifestations of KD in training set mainly included the duration of fever before initial IVIG, conjunctival congestion, red lips/strawberry tongue, enlarged lymph nodes, rash, and stiffness of hands and feet. The median days of fever before the initial IVIG were shorter in the IVIG-resistant group than the IVIG-sensitive group (5 days vs. 6 days, *P* < 0.001), and the incidence of generalized rash was higher (87.2% vs. 71.4%, *P* < 0.05) in the IVIG-resistant group than the IVIG-sensitive group. The conjunctival congestion, redness of lips and tongue, enlarged lymph nodes, and stiffness of hands and feet in children between the two groups did not differ statistically (*P* > 0.05, Table 1).

**TABLE 1:** Demographic features and clinical manifestations between IVIG-sensitive and IVIG-resistant KD children in the training set.

Items	IVIG-sensitive KD	IVIG-resistant KD	Z/x^2^	*P*-value
Patients (n)	588	47		
Gender (M/F)	388/200	30/17	0.090	0.764
Age (month)	23 (12–42)	22 (12–39)	–0.340	0.734
Height (cm)	87.0 (75.0–100.0)	82.0 (78.0–97.0)	–0.476	0.634
Weight (kg)	12.0 (10.0–15.0)	11.5 (9.5–14.5)	–0.730	0.466
BMI (kg/m^2^)	16.0 (14.8–17.5)	15.8 (14.8–18.0)	–0.362	0.717
Fever before IVIG (day)	6 (5–7)	5 (5–6)	–3.738	< 0.001
Conjunctivitis (Yes/No)	501/87	39/8	0.169	0.681
Oral changes (Yes/No)	526/62	44/3	0.820	0.365
Cervical lymphadenopathy (Yes/No)	339/249	21/26	0.097	0.755
Palm edema (Yes/No)	335/253	32/15	2.203	0.138
Rash (Yes/No)	420/168	41/6	5.465	0.019
CAL (Yes/No)	543/45	44/3	0.100	0.751
CKD (Yes/No)	332/256	32/15	2.403	0.121

*IVIG, intravenous immunoglobulin; KD, Kawasaki disease; M/F, male/female; BMI, body mass index; CAL, coronary artery lesions; CKD, classic Kawasaki disease.*

### Laboratory Results Between Intravenous Immunoglobulin-Sensitive and Intravenous Immunoglobulin-Resistant Kawasaki Disease Children in Training Set

In the peripheral blood routine analysis, children with or without IVIG-resistant KD showed significant differences in the following indicators. The IVIG-resistant KD group had significantly lower peripheral blood monocyte (MO)% (3.70% vs. 5.90%, *P* < 0.001), eosinophil (EO)% (0.70% vs. 1.80%, *P* < 0.01) and PNI (46.5 vs. 54.8, *P* < 0.001) than the IVIG-sensitive KD group. In contrast, the IVIG-resistant KD group showed significantly larger peripheral blood NLR (6.4 vs. 4.4, *P* < 0.001) and higher hsCRP (105.0 mg/L vs. 68.2 mg/L, *P* < 0.001) than the IVIG-sensitive KD group. No significant differences were found in total peripheral blood white blood cell, hematocrit, mean corpuscular hemoglobin and hemoglobin (*P* > 0.05). In terms of biochemical parameters, the IVIG-resistant children showed much higher serum alanine aminotransferase (ALT) (59 U/L vs. 22 U/L, *P* < 0.001), aspartate aminotransferase (AST) (42 U/L vs. 29 U/L, *P* < 0.01), total bilirubin (TB) (15.2 μmol/L vs. 7.8 μmol/L, *P* < 0.001), direct bilirubin (DB) (7.2 μmol/L vs. 2.7 μmol/L, *P* < 0.001) and glutamyl transpeptidase (GGT) (106 U/L vs. 27 U/L, *P* < 0.001) than the IVIG-sensitive KD children did. In contrast, the IVIG-resistant KD children showed lower serum prealbumin (PAB) (51.6 g/L vs. 66.6 g/L, *P* < 0.05), sodium ion (Na) (137.1 mmol/L vs. 138.7 mmol/L, *P* < 0.01) and potassium ion (K) (4.3 mmol/L vs. 4.5 mmol/L, *P* < 0.05) than the IVIG-sensitive children. And there was no statistical difference in serum cardiac enzymes (lactate dehydrogenase and creatine kinase-MB) and ALB between the two groups (*P* > 0.05, Table 2).

**TABLE 2:** Blood indicators between IVIG-sensitive and IVIG-resistant KD children in the training set.

Variable(s)	IVIG-sensitive KD	IVIG-resistant KD	Z/x^2^	*P*-value
Peripheral blood WBC (×10^9^/L)	13.38 (10.27–17.52)	14.15 (10.35–21.63)	–1.450	0.147
Peripheral blood NLR	4.4 (2.8–6.9)	6.4 (3.9–11.2)	–7.029	< 0.001
Peripheral blood MO (%)	5.90 (4.40–8.30)	3.70 (2.60–6.00)	–4.857	< 0.001
Peripheral blood EO (%)	1.80 (0.70–3.70)	0.70 (0.20–2.00)	–3.319	0.001
Peripheral blood HCT (%)	33.6 (31.1–35.9)	33.5 (31.5–35.8)	–0.139	0.890
Peripheral blood MCH (pg)	26.4 (25.3–27.3)	26.9 (25.8–27.7)	–1.863	0.062
Peripheral blood Hb (g/L)	106.0 (99.0–114.0)	104.0 (99.0–114.0)	–0.383	0.702
Serum hsCRP (mg/L)	68.2 (37.3–112.0)	105.0 (68.4–168.0)	–3.975	< 0.001
Peripheral blood PNI	54.8 (48.5–61.2)	46.5 (40.6–52.0)	–5.687	< 0.001
Serum ALT (U/L)	22 (12–54)	59 (22–189)	–4.188	< 0.001
Serum AST (U/L)	29 (22–43)	42 (23–82)	–2.841	0.004
Serum ALB (g/L)	38.3 (35.8–41.0)	37.1 (34.0–40.8)	–1.758	0.079
Serum PAB (g/L)	66.6 (47.4–89.2)	51.6 (34.6–81.0)	–2.572	0.010
Serum TB (μmol/L)	7.8 (5.8–11.0)	15.2 (7.9–39.6)	–5.543	< 0.001
Serum DB (μmol/L)	2.7 (2.0–4.4)	7.2 (3.1–27.0)	–5.684	< 0.001
Serum GGT (U/L)	27 (12–97)	106 (18–194)	–4.061	< 0.001
Serum Na (mmol/L)	138.7 (136.8–140.3)	137.1 (134.7–138.8)	–4.154	< 0.001
Serum K (mmol/L)	4.5 (4.0–4.9)	4.3 (3.8–4.7)	–2.076	0.038
Serum LDH (U/L)	287 (242–350)	323 (251–416)	–1.767	0.077
Serum CK-MB (U/L)	26 (20–37)	28 (21–38)	–0.644	0.520

*IVIG, intravenous immunoglobulin; KD, Kawasaki disease; WBC, white blood cell; NLR, neutrophil-to-lymphocyte ratio; MO, monocyte; EO, eosinophil; HCT, hematocrit; MCH, mean corpuscular hemoglobin; Hb, hemoglobin; hsCRP, high sensitivity C-reactive protein; PNI, prognostic nutritional index; ALT, alanine aminotransferase; AST, aspartate aminotransferase; ALB, albumin; PAB, prealbumin; TB, total bilirubin; DB, direct bilirubin; GGT, glutamyl transpeptidase; Na, sodium ion; K, potassium ion; LDH, lactate dehydrogenase; CK-MB, creatine kinase-MB.*

### Conversion of Continuous Variables to Dichotomous Variables in the Training Set

Before performing regression analysis, covariance diagnosis was first performed. Among the 15 variables (rash, duration of fever before IVIG, peripheral blood NLR, PNI, M%, E%, serum ALT, AST, TB, DB, GGT, PAB, Na, K, and hsCRP) which significantly differed between the IVIG-resistant and IVIG-sensitive KD groups as demonstrated by univariate analysis, serum TB and DB had significant covariance and thus the serum DB was deleted based on the frequency of clinical use. Of the remaining 14 variables, all 13 were continuous numerical variables, except for rash which was a categorical variable. For the convenience of the clinical use, the continuous variables were transformed into dichotomous variables based on the cutoff values obtained from the ROC curves. The results were illustrated in Table 3.

**TABLE 3:** The cutoff value of converting continuous variables to dichotomous variables in training set.

Cutoff value	AUC	*P*-value	Sensitivity	Specificity
Fever before IVIG ≤ 5 d	0.658	< 0.001	0.660	0.597
Peripheral blood MO% ≤ 4.65%	0.713	< 0.001	0.638	0.704
Peripheral blood EO% ≤ 0.55%	0.645	0.001	0.468	0.781
Serum hsCRP ≥ 65.8 mg/L	0.674	< 0.001	0.787	0.491
Serum ALT ≥ 37.5 U/L	0.683	< 0.001	0.702	0.663
Serum AST ≥ 37.5 U/L	0.624	0.005	0.596	0.679
Serum TB ≥ 12.8 μmol/L	0.743	< 0.001	0.617	0.827
Serum GGT ≥ 80 U/L	0.678	< 0.001	0.638	0.728
Serum Na ≤ 137.7 mmol/L	0.682	< 0.001	0.638	0.646
Serum K ≤ 4.15 mmol/L	0.591	0.038	0.489	0.701
Peripheral blood NLR ≥ 5.0	0.808	<0.001	0.702	0.813
Peripheral blood PNI ≤ 52.4	0.749	< 0.001	0.809	0.592

*AUC, area under curve; IVIG, intravenous immunoglobulin; KD, Kawasaki disease; MO, monocyte; EO, eosinophil; hsCRP, high sensitivity C-reactive protein; ALT, alanine aminotransferase; AST, aspartate aminotransferase; TB, total bilirubin; GGT, glutamyl transpeptidase; Na, sodium ion; K, potassium ion; NLR, neutrophil-to-lymphocyte ratio; PNI, prognostic nutritional index.*

### Constructing Predictive Scoring Model in the Training Set

The aforementioned 14 variables were incorporated into the binary logistic regression equation, and the backward conditional stepwise regression finally yielded three variables (serum TB, and peripheral blood NLR and PNI) as associated indicators to IVIG sensitivity. The OR (95% CI) values for diagnosing IVIG-resistant KD at serum TB ≥ 12.8 μmol/L were 4.273 (2.149, 8.323), peripheral blood NLR ≥ 5.0 for diagnosing IVIG-resistant KD were 4.761 (2.287, 9.913), and peripheral blood PNI ≤ 52.4 for diagnosing IVIG-resistant KD were 2.478 (1.081, 5.682).

**FIGURE 2:**
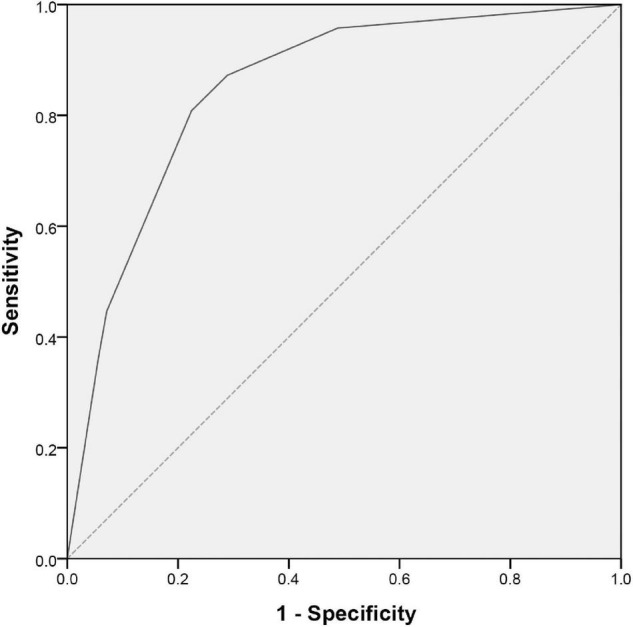
ROC curve for scoring model between IVIG-resistant and IVIG-sensitive KD children. The X and Y axes of the curve represent the predicted false positive rate (1-specificity) and sensitivity, respectively. The dashed line indicates equal true and false positive rates, meaning no predictive value. The solid line is the ROC curve to predict IVIG-resistant KD. A region formed by the ROC curve and the X-axis is called the AUC and its value is 0.850 (95% CI: 0.798–0.901; *P* < 0.001). ROC, receiver operating characteristic; IVIG, intravenous immunoglobulin; KD, Kawasaki disease; AUC, area under the curve; CI, confidence interval.

With the reference to the abovementioned OR values, we defined 2 points for serum TB ≥ 12.8 μmol/L and 0 point for TB < 12.8 μmol/L, 2 points for peripheral blood NLR ≥ 5.0 and 0 point for NLR < 5.0, and 1 point for peripheral blood PNI ≤ 52.4 and 0 point for PNI > 52.4. The constructed predictive scoring model contained the above three indicators, with a total score of 0 to 5 points for each patient. The total score was calculated for each child, and then the ROC curve revealed a cutoff value of three points with an AUC (95% CI) of 0.850 (0.798, 0.901, *P* < 0.001). When the score was ≥3 points, the sensitivity of this scoring model was 80.9% and the specificity was 77.6% in predicting IVIG-resistant KD (Figure 2 and Table 4).

**TABLE 4:** Coefficients of binary logistic regression in training set.

Variable(s)	*P*-value	Odds ratio (95% CI)	Point
Serum TB ≥ 12.8 μmol/L	< 0.001	4.273 (2.149, 8.323)	2
Peripheral blood NLR ≥ 5.0	< 0.001	4.761 (2.287, 9.913)	2
Peripheral blood PNI ≤ 52.4	0.032	2.478 (1.081, 5.682)	1

*CI, Confidence Interval; TB, total bilirubin; NLR, neutrophil-to-lymphocyte ratio; PNI, prognostic nutritional index.*

### Validation of Scoring Model by Real Clinical Diagnosis in External Validation Set

To validate applicability of our scoring model, we calculated the points for each child in the validation set, and constructed a four-grid table (Table 5) based on the actual clinical diagnosis and the points of the predictive scoring model. The predicted diagnosis by the scoring prediction model was then compared with the actual clinical diagnosis of the patient, and the sensitivity, specificity, and accuracy of the predictive scoring model for identifying IVIG-resistant KD were 72.7%, 84.9%, and 83.9%, respectively. We also validated the scoring model for predicting IVIG therapeutic response in CKD and IKD cases separately in the validation set. We found that the sensitivity and specificity of this scoring model to predict the IVIG-resistant KD were 73.3% and 83.9% in CKD patients, respectively; while in IKD cases, the sensitivity and specificity were 66.7% and 88.2%, respectively.

**TABLE 5:** Validation of the scoring model.

Score (point)	Clinical diagnosis		Total
	IVIG-resistant KD	IVIG-sensitive KD	
≥3	24	54	78
<3	9	304	313
Total	33	358	391

*IVIG, intravenous immunoglobulin; KD, Kawasaki disease.*

## Discussion

In our training and validation studies in the central (Wuhan) and northern (Beijing) areas of China, we developed a predictive scoring model for the early IVIG-resistant KD prediction. The predictive scoring model consisted of three variables, serum TB and peripheral blood NLR and PNI. Among them, serum TB ≥ 12.8 μmol/L was scored by 2 points, peripheral blood NLR ≥ 5.0 scored 2 points and peripheral blood PNI ≤ 52.4 scored 1 point, with the total score being 5 points. The sensitivity of the prediction of IVIG-resistant KD was 80.9% and the specificity was 77.6% when a child had a total score of ≥3 points, which was externally validated at different area of China.

Serum TB was scored 2 points in the predictive scoring model and was shown to be an independent predictor. The higher level of TB in the IVIG-resistant KD group was also consistent with previous studies (25). However, the mechanisms by which serum TB was significantly increased in IVIG-resistant KD children have been unclear (32). IVIG-resistant KD has a severe immune-mediated inflammation of the blood vessels, which resulted in multiple organ damage including the liver function injury (33). We showed that serum ALT was more significantly elevated in IVIG-resistant KD than that of IVIG-sensitive KD children (*P* < 0.001), suggesting that IVIG-resistant KD children had severer liver function injury than those with IVIG-sensitive KD.

The peripheral blood NLR score was 2 points in the predictive scoring model. In clinical practice, some children with KD presented with a markedly elevated neutrophils and a decreased lymphocytes in number, and thereby a markedly elevated peripheral blood NLR. In the study, we revealed that children with IVIG-resistant KD had significantly higher peripheral blood NLR than those with IVIG-sensitive KD (6.4 vs. 4.4, *P* < 0.001). Peripheral blood routine indicators such as leukocytes, neutrophils and lymphocytes fluctuate in response to various factors such as inflammation and single indicators are highly variable and non-specific. However, the ratios of the above indicators could better reflect to some extent the inflammatory response (21). IVIG-resistant KD patients had a severe vascular inflammation compared to the IVIG-sensitive KD ones (18). In the study, we revealed that the peripheral blood NLR was an important variable in predicting IVIG resistance.

PNI was an index for nutritional assessment and risk prediction established by Japanese scholars Onodera et al. and was initially applied to the assessment after gastric and intestinal surgery (31). PNI also reflects the inflammatory condition of the body to some extent (34). Moreover, it has also been shown that low pre-treatment PNI levels (PNI < 55) can be used as an adjunctive predictor of CAL (35). In this scoring model, peripheral blood PNI was scored 1 point, where peripheral blood PNI consisted of two indicators, namely serum ALB and peripheral blood lymphocytes. It has been noted that low ALB levels were used in predicting IVIG-resistant KD (36). Another component of PNI is lymphocytes, a simple parameter that reflects the body’s immune response and studies have shown significantly lower lymphocyte counts in IVIG-resistant KD patients (37, 38). However, the absolute lymphocyte value itself is influenced by the total number of leukocytes. Therefore, peripheral blood PNI would be useful to predict IVIG-resistant KD.

Our study successfully constructed a useful scoring model to predict IVIG-resistant KD patients consisting of three indicators: serum TB, peripheral blood NLR and PNI. This predictive scoring model is simple and easy to use and inexpensive. Whether a predictive scoring model can be externally validated in other medical institution is an important factor for the evaluation. The Japanese Egami or Kobayashi scoring model was the currently accepted scoring model with high sensitivity and applicability in Japanese children (16, 17). However, Song et al. found that the predictive sensitivity for IVIG-resistant KD patients were unsatisfactory when the above two scoring models were used in children in Beijing, China (39). While, the predictive scoring model set up in the present study exhibited a high sensitivity and specificity in the validation set in another city in China, suggesting that our scoring model might have a good generalizability.

However, the present study also had some limitations. As a retrospective study, it would have a selective bias of the subjects. Therefore, a prospective randomized and controlled study is needed in the future to assist in predicting early optimization in children with IVIG-resistant KD.

## Conclusion

Early identification and prediction of IVIG-resistant KD patients are extremely necessary in clinical pediatrics. In this study, we successfully constructed a helpful easy-to-perform and inexpensive predictive scoring model in different centers with a satisfactory predictive ability for IVIG-resistant KD cases. This model would be useful in assisting pediatricians in predicting the IVIG-resistant KD patients so as to provide reasonable therapeutic strategy for KD in children.

## Data Availability Statement

The raw data supporting the conclusions of this article will be made available by the authors, without undue reservation.

## Ethics Statement

The studies involving human participants were reviewed and approved by the Ethics Committee of Wuhan Children’s Hospital and Peking University First Hospital. Written informed consent from the participants’ legal guardian/next of kin was not required to participate in this study in accordance with the national legislation and the institutional requirements.

## Author Contributions

JD, HJ, and YZ conceived and designed the project and directed the revision of the manuscript. CL, SW, YL, YS, JF, and DZ acquired the data. YYS, HY, and QZ directed the statistical methods, analyzed and interpreted the data. All authors wrote the manuscript, critically reviewed the manuscript, and approved the final version, access to the primary data and were responsible for the accuracy and completeness of the results.

## Conflict of Interest

The authors declare that the research was conducted in the absence of any commercial or financial relationships that could be construed as a potential conflict of interest.

## Publisher’s Note

All claims expressed in this article are solely those of the authors and do not necessarily represent those of their affiliated organizations, or those of the publisher, the editors and the reviewers. Any product that may be evaluated in this article, or claim that may be made by its manufacturer, is not guaranteed or endorsed by the publisher.
